# Notes and News

**Published:** 1890-11-15

**Authors:** 


### Notes and News.

Collected and Communicated.

The Bradford Board of Guardians have got to enlarge their infirmary, and the question arises—how? By the pavilion system, undoubtedly, answers the Local Board inspector, and we hope his advice will be taken. It is a pity that the extension of the Liverpool Infirmary, being in block form and highly commended, has, for the time being, rather thrown discredit on the pavilion system.

Mrs. Garrett Anderson means to procure a new lease of life for old ladies by teaching them to play ball; General Booth is going to cure destitution by means of his big drum; the Fabian Society is going to put us all on a happy level through municipal socialism. It is delightful to think that there is still so much enthusiasm amongst the children of men, and we hope some results will follow. Certainly "In Darkest England" is the most interesting book that has been published for many a long day, and should be studied by every practical philanthropist. But even supposing the scheme carried out and the slums cleared, would not the country yokels and foreign Jews soon drift into swept and garnished London, and the second state of this unhappy city be worse than the first?

It is somewhat astonishing to find that the Liverpool Hospital Sunday Fund is acutely sensitive to the state of trade; this, and other facts of interest, are made apparent in the current report. The total amounts of the Sunday fund for 1890 as compared with the previous year were as follows: Collections, £6,949 in 1890, as against £6,766 in 1889, showing an increase of £183. The bank interest this year amounted to £43, as against £37 last year; and the donations this year reached £70, as against £84 the previous year. The total sum received this year amounted to £7,063, as against 6,888 the previous year, the increase being £175. The expenses amounted to £91, being a slight increase in the expenses on last year. The net sum available for distribution was £6,971, and the actual grants made from the fund to the medical charities of Liverpool was £10,600. The Salvation Army appears amongst the subscribers for the first time.

Sheffield Infirmary report is always a clear and concise document, and always has a good record of work done to give. The accounts are accurate and exhaustive, and include the "unproductive investments" which the House of Lords' Committee failed to find in some of the reports submitted to them. The house surgeon also supplies an interesting summary of the cases treated during the year, giving the result of each case. The house-keeping seems to be economically done. The number of beds now available is 200, of which 44 are devoted to children. The expenditure last year was £10,837.

Dr. G. de G. Griffith, of the Zenana College, has received the following letter: "Dear Sir,—Allow me to introduce myself to you as the brother of your new scholar, Miss Rebecca Krikorian, of Aintab, Turkey. First of all, I wish to express to you our gratitude—that is, my father's and mine—for the kindness you have shown in receiving her to your institution. We appreciate it very highly, and we are sure that the medical instruction she is going to receive there will be of great use here among our people. The good work which such a lady physician as Mrs. Shepard is doing among Mohammedan women, as well as among the Christian women, is of such great and wide importance that all the people of every class appreciate it very highly. So we hope that your institution will add another labourer to enlighten the minds and lighten the burden of the women of this benighted land. We hope and are sure that your fatherly care will be upon Miss Rebecca. Please to accept the Christian love of her father, Pastor Kara Krikorian, and the best Christian regards of yours, most respectfully, Hohannes Krikorian." Four more Armenian ladies are anxious to come over and study medicine.

Chester Guardians are going to indulge in the expensive luxury of a Government Local Board Inquiry into the recent scandal. It will be remembered that the inspector found a small patient very ill and with sores not properly dressed. The medical officer has been censured, but evidently the guardians do not consider this measure of the local board strong enough; they want to call on Dr. Harrison to resign. At the meeting on November 4th it was very notable how the guardians lost sight of the question of neglect, and dropped to the discussion of the manners of the doctor, and the fact that he did not touch his hat to the guardians. Here is the sort of speech that was made: "Mr. T. Browne: It is very little use of our being guardians of this Union; if we cannot choose the dancing, what is the use of paying the piper? It has been passed by this Board that the doctor send in his resignation. If he had treated the guardians in the same way as he has written to the Local Government Board, he would have been treated as a gentleman by this Board—(hear, hear)—but he would not condescend to answer the questions put to him. He treated us as vastly inferior to himself, and took no notice of the remarks made. If he had only said he was sorry on that morning, I feel that everyone round this Board was generous enough to have looked over any misconduct had he only treated the Board with the respect it deserves. (Hear, hear.)" Now this is where Boards of Guardians go wrong; they have not sufficiently studied the story of the three tailors of Tooley Street—"We, the people of England"; nor do they recognise sufficiently the difference between a public and a private servant. Thus an infirmary medical officer often has to put up with much annoyance, and squabbles arise with very little justification. On the other hand we acknowledge that it is a very thankless task to be a poor-law guardian, and that with the gentleman, be he guardian or doctor, ever lies the duty of courtesy to all.

The following vacancies are announced this week, the amount of the salary in each case being given after the name of the institution:—

**Resident Medical Officers.**

Colney Hatch Asylum, Fifth Assistant Medical Officer—£120, board, &c.
Alnwick Infirmary, House Surgeon—£120, rooms, &c.
Manchester Hospital for Sick Children, Medical Officer—£80, board, &c.
Leicester Infirmary, Assistant House Surgeon—£80, board, &c.

**Non-Resident Medical Officers.**

Edinburgh Deaf and Dumb Institution, Oculist and Aurist.
Birmingham Royal Orthopædic Hospital, Honorary Surgeon.
Bradford Friendly Societies, Out-door Assistant—£120.
Penrith Union, Medical Officer—£38.
University College, London, Jodrell Professor of Anatomy and Zoology.
University College, London, Curator of Anatomical Museum—£150.
London Hospital, Surgical Registrar—£100.

An American contemporary reports that half a drachm of chloride of ammonium will restore to a drunken person the use of his faculties. It is to be hoped that confirmed inebriates will not be tempted by the knowledge of this to pervert its use so as to enable them to indulge still further in their unfortunate propensity.

Reigate and Redhill Cottage Hospital supporters held their annual meeting in the end of October. The twenty-fourth annual report of the committee stated that there had been 210 patients under treatment during the year, as against 192 in the previous year. The results were: Cured or convalescent, 159 (or 79·1 per cent. of completed cases); relieved, 36; not benefited, 3; died, 3 (or 1·4 per cent.); remaining in hospital, 9. The average duration of each case was 22·6 days. The committee thought it would be desirable to vote a further sum at the annual meeting for the purpose of contributing to the seaside convalescent institutions, in order that they might have a bed at their disposal for patients requiring change of air. £30 was voted.

Derbyshire Hospital for Sick Children held its annual meeting on November 4th, when Mr. Seale Haslam took the chair as President for the ensuing year. During the year 2,334 patients were treated, namely, 316 in and 2,018 out, as compared with 1,507 in the previous year, or an increase of 829. The ordinary current expenses for the year were £670 0s. 11d., or £48 11s. 1d. less than in the previous year; but although every effort had been made by the Board to keep down the expenses, the deficiency on this account at the end of the year was £211 13s. 3d., or £17 8s. 8d. more than last year. This state of affairs is caused by the great increase in the number of patients and in the size of the institution, as compared with the old premises on the Duffield Road, and to the fact that the contributions by the public during the last few years to the revenue account have not increased in proportion.

It is impossible to delay longer a reference to the Birkenhead Hospital scandal, though probably a calm statement of the case will not be arrived at till the present epidemic and ill-feeling has subsided. The Fever Hospital was left in the charge of an old soldier and his wife, both untrained; an epidemic of typhus broke out, and two nurses volunteered to go and aid at the Fever Hospital; they found the place in a fearful state, too shocking to describe, and they set to work to scrub the floors as well as nurse the numerous patients. They both took typhus, and one has since died. Three of the doctors attending the hospital have taken typhus. The second batch of nurses sent refused to stay unless certain sanitary defects were remedied and nursing appliances supplied. Now the local papers are full of the subject, and the typhus is spreading, and the end of the scandal remains to be seen.

Systematic cleaning out and lime washing the hot air flues have had a most beneficial effect upon the health of the inmates of Pentonville prison. According to the report of Dr. Innes, the medical officer to the prison, erysipelas is now of much less frequency, and pneumonia, which had previously proved fatal in so many cases, has been completely eradicated.

The ladies of Worcester did very well with their first Hospital Saturday collection, for it amounted to £181, and the ladies have expressed their willingness to a similar collection next year. An analysis of the collection shows that it included 11 sovereigns, 24 half-sovereigns, 1 crown, 1 four shilling piece, 50 half-crowns, 110 florins, 659 shillings, 1,276 sixpences, 703 threepenny bits, 11,646 pennies, 2,616 halfpennies, 19 farthings, 2 penny stamps, 1 watch key, 1 French penny, 1 undistinguishable copper coin.

This is what Dr. Ephraim Cutler thinks about cookery. He calls cooks "queens in our kitchen, who govern the whole house. The foods prepared by them form the staple of our lives. If we have poor food we are not well; if we have good food properly cooked, then our lives run smoothly and in health. The evils in strong drink ar e great, but the evils of bad cooking and ill-selected food are greater. Would that the time spent by the queens of our parlours on crazy quilts, screens, and fancy needlework (well enough in their place), were given to the beautiful problems connected with food that are so vitally important and constantly pressing on our attention every time we eat." "Count Rumford," says Dr. Cutler, "was cook and philosopher; this union of offices did not degrade him. Nor is it degrading to be a cook, Mrs. Grundy notwithstanding!"

